# Cardiovascular Autonomic Nervous System in a Patient With Hereditary Angioedema Affected by COVID-19

**DOI:** 10.7759/cureus.56449

**Published:** 2024-03-19

**Authors:** Beatrice De Maria, Monica Parati, Yagis Bey, Laura Adelaide Dalla Vecchia, Francesca Perego

**Affiliations:** 1 Department of Internal Medicine, Istituti Clinici Scientifici Maugeri IRCCS (Istituto di Ricovero e Cura a Carattere Scientifico), Milan, ITA

**Keywords:** blood pressure variability, baroreflex sensitivity, heart rate variability analysis, hereditary angioedema without normal c1 inhibitor, covid-19

## Abstract

Autonomic nervous system (ANS) regulation in hereditary angioedema (HAE) and coronavirus disease 2019 (COVID-19) is unknown. ANS alterations could be manifested during both the acute and post-acute phases of COVID-19. Implications of acute and chronic inflammation on ANS in HAE need to be addressed. In this case report, we monitored the systolic arterial blood pressure variability and baroreflex sensitivity in a female HAE patient both before experiencing COVID-19 symptoms and one month afterward. We also tracked the heart rate variability on the day preceding symptom onset, the day of symptom onset (SYM), the day following SYM, five days after SYM, the day of the first negative nasopharyngeal swab (i.e., 12 days after SYM), and one month after symptom onset. The results of this case report provide the characterization of vascular and cardiac autonomic profiles in an HAE patient until the resolution of an acute infection, a potential trigger for the acute HAE attack.

## Introduction

The respiratory system is the main target of COVID-19 but multiorgan damage is frequently described [1]. Symptoms such as smell and taste loss, focal neurological signs, fatigue, and lightheadedness are reported in more than one-third of patients and can be attributed to the central and autonomic nervous system (ANS) during both the acute and recovery phases characterizing the so-called post-COVID-19 syndrome [1]. Among them, orthostatic hypotension and intolerance, postural orthostatic tachycardia syndrome, and heart rate variability (HRV) impairment are commonly observed [1,2]. A recent systematic review [1] also demonstrated the involvement of the ANS at the cardiovascular level as a predictor of the prognosis during and after COVID-19 infection.

Hereditary angioedema (HAE) due to C1-inhibitor (C1-INH) deficiency is a rare genetic disorder caused by a mutation in the SERPING1 gene, with an estimated prevalence of 1:50,000 worldwide. Diagnosing HAE involves measuring serum or plasma levels of C1-INH function, which may be reduced or normal, C1-INH protein levels, as well as complement C4 levels, which are always severely reduced (less than 50% of the lower normal level) [1].

HAE is characterized by recurrent, sudden, and transient swelling triggered by the release of bradykinin, which occurs via the activation of components of the kallikrein-kinin system implicated in inflammatory responses [3,4]. Bradykinin binds to the bradykinin B2 receptors and stimulates their activation, thereby mediating vasodilation and increased capillary permeability [5,6]. The disease presents with recurrent swelling attacks involving the extremities (hands and feet), face (eyes, lips, and tongue), genitals, gastrointestinal tract (painful abdominal cramps), and laryngeal edema that could lead to suffocation in a few minutes or hours if the attacks are not properly treated [7]. Based on this observation, the current guidelines for HAE treatment recommend that all patients possess and keep on hand on-demand medication to treat a minimum of two attacks [3].

HAE attacks are known to be triggered by a variety of conditions and events. Trauma, dental, medical, and surgical procedures may precede an attack. Medications such as oral contraceptives containing estrogen, estrogen hormone replacement therapy, and angiotensin-converting enzyme (ACE) inhibitor antihypertensive agents might escalate the frequency of HAE symptoms and hence should be rigorously avoided by patients. Common triggers, including psychological and physiological stress, could have a role. Febrile illness, acute infections, such as COVID-19, and inflammatory conditions are clear examples of physiological stress, a potent risk factor for the development of the attack. Indeed, all HAE patients should be educated on the triggers that could potentially provoke attacks. In this case, there is no clear suggestion for the use of short-term prophylaxis to avoid attacks in patients with ongoing on-demand or long-term prophylactic treatment [3].

The COVID-19 pandemic has raised many questions and created uncertainty among patients with HAE and their healthcare providers. Questions are primarily related to the presence of possible different patterns of the infection, including the contagion risk, the possibility of a different disease course, the impact on the activity of HAE disease, the possible interaction between COVID-19, and the therapies approved for the treatment of the HAE acute attacks and also the efficacy of prophylactic treatment during the acute phase of the infection. The impact of COVID-19 vaccines on individuals with HAE remains an area that requires further investigation. To date, there are available data that provide partial insights into these inquiries and address some concerns. Previous studies indicated no significant increase in HAE attacks during or after COVID-19, although a few patients relying on on-demand therapy reported heightened HAE activity [8,9]. There is no indication that HAE patients faced an increased risk of infections or adverse effects due to the vaccine.

In a survey conducted in the Netherlands, involving 111 COVID-19 vaccine doses administered to HAE patients, 11 attacks were documented, most of which were managed with on-demand medication. The majority of these attacks occurred in patients with poorly controlled disease [10].

However, there is no information regarding the pathological or physiological functioning of cardiovascular regulation in HAE patients during and after the acute phase of COVID-19. Even if the involvement of the ANS in COVID-19 is recognized, it is still not known how the involvement of the ANS progresses over time during the infection. In addition, the evaluation of the cardiovascular ANS in a patient with HAE infected by COVID-19 has never been described [9,11].

The ANS is among the modulators of the vascular tone that can be studied through the analysis of arterial pressure (AP) variability, thereby offering indices related to the sympathetic branch of vascular neural control [12]. Furthermore, analyzing the variability of RR intervals derived from continuous electrocardiogram (ECG) monitoring provides non-invasive indices related to the vagal branch of the cardiac neural control [13], which are known to carry prognostic significance in several cardiac diseases [14] and are associated with the severity of COVID-19 symptoms [15]. Bivariate analysis of RR and systolic AP (SAP) provides information about the functioning of the baroreflex (BR), one of the main physiological control mechanisms involved in the maintenance of the homeostasis of the AP [16]. The cardiac BR reacts to AP changes modulating the RR: an increase in the AP physiologically determines an increase of the RR, while a decrease in the AP determines a decrease of the RR. The functioning of the BR is usually estimated by the BR sensitivity (BRS), i.e., the response of the RR to a unit variation of the systolic AP [16].

The aim of the present study is the description of the ANS modulation during COVID-19 infection in an HAE patient.

This article was partially presented as meeting abstracts [17,18] at the 13th edition of the Biennial International Scientific Conference on C1-Inhibitor Deficiency and Other Bradykinin-Mediated Angioedema that took place from 4 to 7 May 2023 and at the European Academy of Allergy and Clinical Immunology Congress that took place from 9 to 11 June 2023.

## Case presentation

We describe the case of a female HAE patient (58 years, BMI: 29.98 kg/m^2^) diagnosed when she was eight years old. During her life, she experienced laryngeal attacks leading to recurrent hospitalization. Throughout her life, she had a sustained severe form of HAE, experiencing more than two attacks per month. In the past, she underwent long-term prophylactic treatment with attenuated androgens before transitioning to lanadelumab [19], a fully human monoclonal antibody of plasma kallikrein, administered subcutaneously once per month for the last five years, effectively controlling the disease with less than one attack per year.

The patient underwent a head-up-tilt-test the day prior to the onset of fever attributed to COVID-19 (PRE). Coincidentally, as part of a research protocol screening visit focusing on the study of the ANS in patients with angioedema, approved by the Ethics Committee of Istituti Clinici Scientifici Maugeri IRCCS in Pavia (Italy) with approval granted on May 14, 2019, under approval number CE2303, a multiday Holter ECG was recorded at PRE, followed by recordings on the day of symptom onset (SYM), the first day after SYM (SYM1), five days after SYM (SYM5), the day of the negative nasopharyngeal swab (i.e., 12 days after SYM, SYM12), and one month after symptom onset (SYM30). The head-up-tilt test was repeated at SYM30.

The head-up tilt test is a provocative test applied to study the reaction of the cardiovascular ANS to passive orthostasis. The maneuver is expected to enhance the sympathetic branch and induce a reduction in the activity of the vagal branch. The test involves recording continuous ECG (Lab3, Marazza, Monza, Italy) and non-invasive AP (Finometer Midi, Finapres Medical System, Enschede, Netherlands) for 10 minutes while in a supine position (REST), followed by rotating the tilting table to 70° and continuing to record the signals for another 10 minutes (TILT). The sampling rate of the signals was 1000 Hz.

The test was performed in the morning, in a quiet room with a comfortable temperature. The patient was asked to avoid caffeinated and alcoholic beverages in the 24 hours preceding the test and to avoid physical exercise.

The signals recorded during the test are processed to derive quantitative indices helpful in the characterization of the cardiovascular ANS. The first step involves extracting the beat-to-beat time series from the analog signals. The ECG produces an RR time series, where RR signifies the temporal interval between two successive R peaks detected on the ECG. Similarly, the AP signal yields the SAP time series, where SAP represents the maximum arterial pressure within each RR interval. Segments of 300 continuous samples of RR and SAP series were selected during REST and TILT for further analysis. The mean of RR and SAP series (µ_RR_ and µ_SAP_) were calculated. µ_RR_ was expressed in ms, while µ_SAP_ was in mmHg. Parametric power spectral analysis was then performed over both the RR and SAP series: the absolute power of the RR series in the high-frequency band (HF, 0.15-0.40 Hz) was taken as an index of the vagal cardiac modulation (HF_RR_) while the absolute power of the SAP series in the low-frequency band (LF, 0.04-0.15 Hz) provided information about the vascular sympathetic modulation (LF_SAP_) [12]. HF_RR_ was expressed in ms^2^, while LF_SAP_ was in mmHg^2^. To evaluate the contribution of the baroreflex (BR), cardiac baroreflex sensitivity (BRS) in the low-frequency (LF) range (0.04-0.15 Hz) was computed using the spectral method [16] and expressed in ms/mmHg^-1^.

The multiday Holter was performed by recording the ECG using a portable device (Faros 360°, Bittium Corporation, Oulu, Finland; Sylco srl, Monza, Italy) during the regular daily and nocturnal activities. The sampling rate was 500 Hz. The RR series was also derived from the ECG signal. Selection of 5000 continuous samples during the night-time (from 1 a.m. to 5 a.m.) was performed for each of the considered days of recording (i.e., PRE, SYM, SYM1, SYM5, SYM12, and SYM30) for further analysis. On these selections, µ_RR_ was calculated, and an iterated parametric power spectral analysis over windows of 250 RRs with an overlap of 200 RRs was performed over the RR series. The median of the distribution was considered representative. The normalized HF_RR_ was utilized as an indicator of cardiac vagal modulation (HF_RR_) [12].

**Figure 1:**
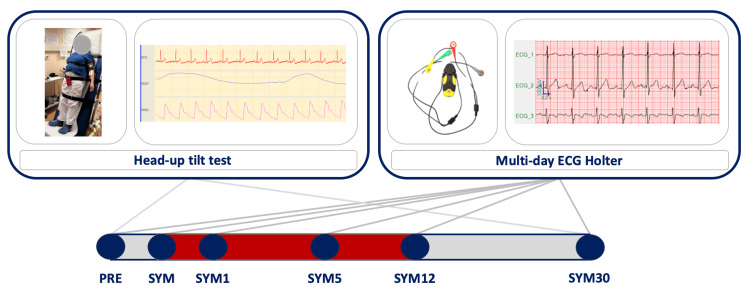
Schematic representation of the experimental set-up and assessment timeline. Schematic representation of the experimental set-up used during head-up tilt test and multiday Holter ECG and of the assessment timeline including the day prior to the fever onset due to COVID-19 (PRE), symptom onset (SYM), the day after symptom onset (SYM1), five days after symptom onset (SYM5), 12 days after symptom onset (SYM12), and one month after symptom onset (SYM30). The red bar indicates the period of COVID-19 infection.

A schematic representation of the experimental set-up and assessment timeline is reported in Figure 1.

During the one-month observation period, the patient did not experience any HAE attacks. Symptoms of COVID-19 consisted of fever (up to 39°C) and muscle discomfort, which persisted for five days without requiring hospitalization. The treatment of COVID-19 symptoms included paracetamol and anti-inflammatory drugs.

The findings from the head-up tilt protocol are reported in Table 1 and indicated that µ_RR_ decreased by 84 ms from REST to TILT in PRE and by 127 ms in SYM30, while µ_SAP_ decreased by 10 mmHg from REST to TILT in PRE but remained stable in SYM30. A physiological reduction in HF_RR_ was observed in both PRE (from 254 to 123 ms^2^) and SYM30 (from 275 to 88 ms^2^). Conversely, the physiological increase of LF_SAP_ during TILT compared to REST was not observed in PRE (decreasing from 1.14 to 0.58 mmHg^2^), whereas it was present in SYM30 (increasing from 4.17 to 5.44 mmHg^2^). The expected decrease of BRS with TILT was absent, showing a paradoxical increase in PRE (from 5.26 to 14.52 ms/mmHg^-1^), but it was restored in SYM30 (from 2.16 to 1.20 ms/mmHg^-1^).

**Table 1:** Indices of sympathetic vascular neural modulation and baroreflex sensitivity indices during the head-up tilt test. Results of the sympathetic vascular neural modulation and baroreflex sensitivity indices obtained during the supine resting condition (REST) and during the head-up tilt test (TILT) prior to the onset of fever attributed to COVID-19 (PRE) and one month after symptom onset (TILT). HF: high frequency; LF: low frequency; SAP: systolic arterial pressure; BRS: baroreflex sensitivity.

	PRE		SYM30	
	REST	TILT	REST	TILT
µ_RR_ (ms)	1033	949	987	860
µ_SAP_ (ms)	120	110	132	130
HF_RR_ (ms^2^)	254	123	275	88
LF_SAP _(mmHg^2^)	1.14	0.58	4.17	5.44
BRS (ms/mmHg^-1^)	5.26	14.52	2.16	1.20

**Figure 2:**
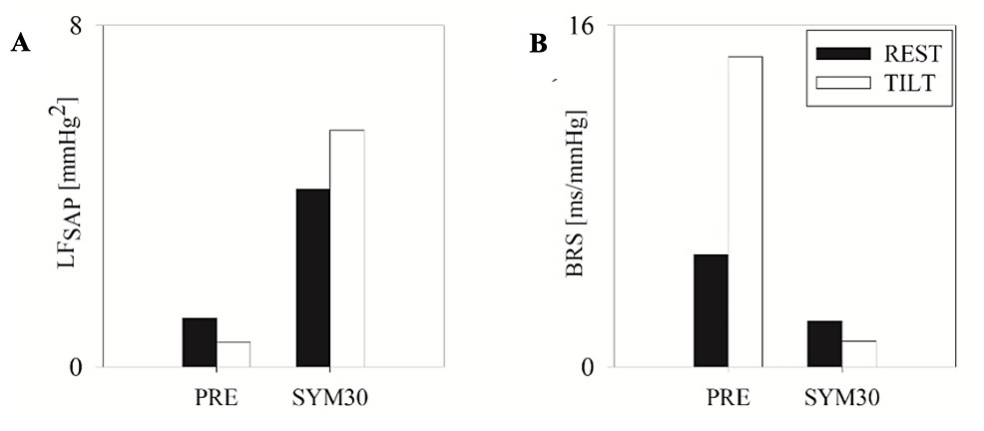
Sympathetic vascular neural modulation and baroreflex sensitivity during the head-up tilt test. Sympathetic vascular neural modulation (panel A) and baroreflex sensitivity (panel B) were assessed the day prior to fever onset due to COVID-19 (PRE) and one month after symptom onset (SYM30) in the supine position (REST, represented by black bars) and during the head-up tilt test at 70° (TILT, represented by white bars).

 The main findings are summarized in Figure 2 (black bars represent REST, while white bars represent TILT).

**Figure 3:**
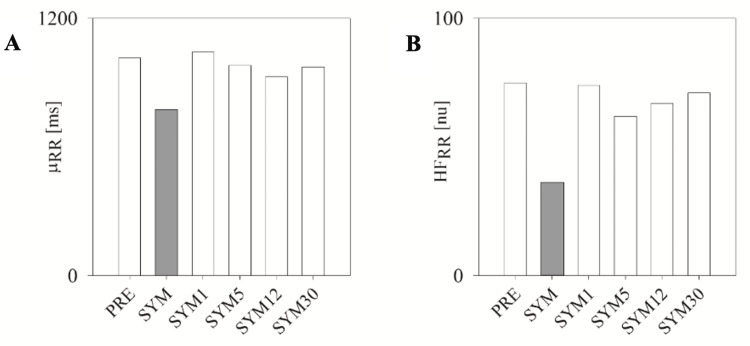
Cardiac neural control index and vagal cardiac modulation from multiday Holter ECG. Cardiac neural control indices (mean of the RR series (A); vagal cardiac modulation (b) estimated from the multiday Holter ECG on the day leading up to fever onset due to COVID-19 (PRE), symptom onset (SYM), the day after symptom onset (SYM1), five days after symptom onset (SYM5), 12 days after symptom onset (SYM12), and one month after symptom onset (SYM30).

The outcomes from the multiday Holter ECG monitoring are depicted in Figure 3 (grey bars represent SYM). In comparison to PRE, both µ_RR_ (775 vs. 1016 ms) and HFRR (36 vs. 75 ms^2^) were reduced during SYM. However, all the mentioned parameters reverted to values similar to PRE during the subsequent days (i.e., SYM1, SYM5, SYM12, and SYM30 days).

## Discussion

This case report marks the first instance of concurrent recording and longitudinal monitoring of the vascular and cardiac autonomic profile in an HAE patient until the resolution of an acute infection, specifically an inflammatory condition, which could serve as a potential trigger for an HAE acute attack. This holds significance for patient management, particularly concerning the unresolved question regarding short-term prophylactic approaches in patients exposed to HAE triggers and undergoing long-term prophylaxis.

This case report proposes a comprehensive assessment of the ANS during orthostatic stress in a controlled test condition and prolonged heart rate variability monitoring during daily life.

Regarding the head-up tilt test results, the decrease in µ_RR_ during TILT compared to REST was evident at both time points (PRE and SYM30) reflecting an increase in the heart rate in response to the orthostatic challenge. Notably, the variation was most pronounced and closest to the physiological value (i.e., 10 bpm) during SYM30. In addition, a decrease in µ_SAP_, a lack of increase of LF_SAP_, and a lack of decrease of BRS in response to TILT were observed in PRE. These findings collectively indicate that during the prodromal phase of COVID-19, the autonomic profile of this HAE patient was characterized by the lack of activation in the vascular sympathetic branch and baroreflex dysfunction during a head-up tilt test, which was not linked to the occurrence of acute angioedema attacks. Regarding cardiac neural control, the response was present but more attenuated during PRE compared to SYM30, consistent with a lower increase in heart rate in response to the orthostatic challenge during PRE compared to SYM30.

Regarding multiday Holter ECG monitoring, throughout COVID-19, the ANS profile in an HAE patient undergoing long-term prophylaxis with lanadelumab displayed diminished cardiac vagal modulation solely on the initial day of symptom onset, without any subsequent alterations observed during the day preceding symptom onset, the progression of the illness, or the post-acute phase. This decreased cardiac vagal modulation was reflected by higher heart rate and lower HF_RR_ on the day of symptoms onset.

These results align with the findings of Milovanovic et al. [20], who observed lower HF_RR_ only in milder hospitalized patients. Although our patient had a mild degree of infection severity and was not hospitalized, the impairment of the autonomic balance was still notable. While ANS evaluation has been suggested as a biomarker of severity in other studies, the data are contradictory.

## Conclusions

In this patient, during the pre-symptomatic phase, the vascular component appears to be primarily involved, as expected due to vasodilation and increased vascular permeability frequently induced by inflammation. Repeated measurement of the cardiac autonomic profile ruled out the presence of a prolonged effect of COVID-19 in this patient. Interestingly, despite the observed dysregulation of the vascular autonomic component, it is noteworthy that the acute HAE attack did not manifest, likely attributable to the beneficial impact of long-term prophylactic treatment on plasma kallikrein level regulation.
